# Post-PCI Inflammation and Diastolic Dysfunction in Patients with Metabolic Risk Factors: A Retrospective Observational Study

**DOI:** 10.3390/medicina61112015

**Published:** 2025-11-11

**Authors:** Alexandra Manuela Buzle, Corina Cinezan, Paul Sextil Sasu, Adrian Tudor Cura, Marc Cristian Ghitea, Evelin Claudia Ghitea, Maria Flavia Gîtea, Aura Bianca Luncan, Timea Claudia Ghitea, Mircea Ioachim Popescu

**Affiliations:** 1 Department of Medical Disciplines, Faculty of Medicine and Pharmacy, University of Oradea, 410068 Oradea, Romania; 2 Clinical County Emergency Hospital Bihor, 410169 Oradea, Romania; 3 Faculty of Medicine and Pharmacy, University of Oradea, 410068 Oradea, Romanialuncan.aurabianca@student.uoradea.ro (A.B.L.); 4 Pharmacy Department, Faculty of Medicine and Pharmacy, University of Oradea, 410068 Oradea, Romania; 5 Department of Clinical Discipline, Faculty of Medicine and Pharmacy, University of Oradea, 410068 Oradea, Romania

**Keywords:** diastolic dysfunction, inflammation, CRP, NT-proBNP, ACS, PCI, metabolic risk, high-sensitivity troponin

## Abstract

*Background and Objectives:* Left ventricular diastolic dysfunction (LVDD) is a known precursor of heart failure with preserved ejection fraction (HFpEF), particularly in patients with metabolic comorbidities. Acute coronary syndrome (ACS) and percutaneous coronary interventions (PCI) may exacerbate LVDD via systemic inflammation. This study aimed to explore the association between post-procedural systemic inflammation and the severity of diastolic dysfunction in patients with ACS and metabolic comorbidities. *Materials and Methods:* A retrospective observational study was conducted in 181 patients with ACS who underwent PCI. Inflammatory markers (leukocytes, neutrophils, and C-reactive protein [CRP]) measured at 24–48 h post-intervention were analyzed in relation to diastolic dysfunction, assessed by echocardiography. Multivariable ordinal logistic regression and correlation analyses were performed. *Results:* CRP showed a non-significant trend toward association with more advanced diastolic dysfunction (*p* = 0.081). Hypertension had a positive but nonsignificant coefficient. Other metabolic comorbidities (diabetes, dyslipidemia, and obesity) were not significantly associated. The correlation between N-terminal pro-B-type natriuretic peptide (NT-proBNP) and troponin was exploratory. NT-proBNP was the only marker significantly correlated with high-sensitivity troponin (TrHS) at 48 h, indicating a link between myocardial injury and wall stress. *Conclusions:* CRP may be weakly associated with the severity of diastolic dysfunction post-PCI. However, classical metabolic comorbidities were not independently predictive. Post-PCI inflammation showed only modest, non-significant trends toward diastolic impairment, warranting confirmation in larger prospective studies.

## 1. Introduction

Left ventricular diastolic dysfunction is a key precursor of heart failure with preserved ejection fraction (HFpEF), a condition frequently encountered in patients with metabolic comorbidities such as hypertension, type 2 diabetes, dyslipidemia, and obesity [1,2,3,4]. In the context of coronary interventions, this dysfunction can be exacerbated by acute inflammatory processes triggered by vascular trauma and myocardial ischemia [5,6,7].

Systemic inflammation plays a central role in myocardial remodeling, impairing diastolic relaxation and ventricular compliance [8,9,10]. Biomarkers such as leukocytes, neutrophils, and C-reactive protein (CRP) can reflect the intensity of the inflammatory response and may serve as prognostic indicators in cardiovascular disease [11]. However, the interaction between post-interventional inflammatory status and the degree of diastolic dysfunction remains insufficiently understood, particularly in subgroups with increased metabolic risk [12,13]. However, their relationship with the severity of LVDD in the early post-intervention period remains unclear, especially in patients with multiple metabolic comorbidities [14,15,16,17].

Ultimately, this study seeks to elucidate the relationship between early systemic inflammation following coronary intervention and the degree of diastolic dysfunction in metabolically at-risk patients [18]. It further aims to determine the extent to which inflammatory markers—leukocytes, neutrophils, and CRP—correlate with diastolic impairment, and whether this relationship is modulated by the presence of metabolic conditions such as hypertension, type 2 diabetes mellitus, dyslipidemia, or obesity. A better understanding of these associations could contribute to the early identification of patients at high risk of diastolic dysfunction and inform personalized post-interventional therapeutic strategies. Inflammation plays a central role in post-ischemic myocardial remodeling, influencing both systolic recovery and diastolic relaxation. Following percutaneous coronary intervention (PCI), procedural and reperfusion-related injury triggers an acute inflammatory response characterized by increased circulating C-reactive protein (CRP), leukocytes, and neutrophils. These mediators contribute to transient myocardial edema, oxidative stress, and extracellular matrix expansion, processes that may impair ventricular compliance and relaxation. In patients with metabolic comorbidities such as hypertension, diabetes, or obesity, the pre-existing state of low-grade inflammation and endothelial dysfunction may amplify this acute response, predisposing them to diastolic dysfunction even after successful revascularization. Therefore, assessing inflammatory biomarkers early after PCI may help identify patients at higher risk for post-procedural diastolic impairment and adverse remodeling.

The present study aims to investigate the association between the early post-interventional inflammatory response (measured at 24–48 h) and the severity of diastolic dysfunction in patients with major metabolic risk factors. Furthermore, the analysis explores whether specific combinations of metabolic comorbidities predict a more advanced degree of diastolic impairment in the setting of acute coronary syndrome (ACS) treated interventionally. We hypothesized that elevated post-PCI inflammatory markers would be associated with more advanced diastolic dysfunction in metabolically at-risk patients.

## 2. Materials and Methods

### 2.1. Study Design and Population

This retrospective observational study included patients admitted with acute coronary syndrome (ACS) who underwent interventional treatment in a tertiary-level interventional cardiology unit. A total of 181 patients were consecutively selected based on the availability of complete clinical, biological, and echocardiographic data relevant to the study objectives. Ejection fraction was recorded in all patients, with the majority (73.7%) having preserved systolic function (LVEF ≥ 50%). Patients with reduced EF (<45%) were not excluded, but sensitivity analyses did not show significant differences in the direction of associations when stratified by EF. Nevertheless, reduced EF may represent a confounding factor, as diastolic dysfunction can coexist with impaired systolic function.

### 2.2. Inclusion and Exclusion Criteria

Patients were eligible if they met the following inclusion criteria:

- Age ≥ 18 years;
- Diagnosis of ACS confirmed by coronary angiography;
- Undergoing percutaneous coronary intervention (PCI);
- Availability of post-interventional data: complete blood count (including leukocytes and neutrophils), CRP, and echocardiographic assessment of diastolic function.

Exclusion criteria were as follows:

- Presence of systemic infections at admission or prior to hospitalization;
- Active autoimmune, hematologic, or malignant diseases;
- Incomplete or failed interventional procedures;
- Missing essential data related to diastolic function or inflammatory markers;
- Ongoing chronic immunosuppressive therapy.

### 2.3. Data Collection

Clinical and paraclinical data were extracted from electronic medical records (Hospital Information System, Medis^®^, version 5.2, Bucharest, Romania) and patient observation sheets. The following variables were recorded:

Demographics: age, sex, and residential area (urban/rural).

Metabolic comorbidities: hypertension, type 2 diabetes mellitus, dyslipidemia, and obesity (clinically defined).

Diagnoses were based on medical records and/or ongoing treatment with antihypertensives, glucose-lowering, or lipid-lowering drugs.

Renal function (eGFR) and LVEF were included in sensitivity analyses; detailed medication data were unavailable and acknowledged as a limitation.

Lifestyle factors: smoking status and alcohol consumption.

Inflammatory markers: leukocytes and neutrophils (at admission, post-intervention, and 48 h), CRP (mg/dL), erythrocyte sedimentation rate (ESR, mm/h), and fibrinogen (mg/dL).

Echocardiographic parameters: diastolic dysfunction grade (0 to 3) and left ventricular ejection fraction (LVEF).

Cardiac biomarkers: high-sensitivity troponin (HS-Tn) and N-terminal pro-B-type natriuretic peptide (NT-proBNP).

Clinical risk scores: TIMI and Killip classification.

Hematological and biochemical parameters, including leukocytes, neutrophils, CRP, ESR, and fibrinogen, were analyzed using automated laboratory systems (Sysmex XN-1000™, Sysmex Corporation, Kobe, Japan) and a Cobas c501 analyzer (Roche Diagnostics GmbH, Mannheim, Germany) according to standard hospital protocols.

Cardiac biomarkers—high-sensitivity troponin (HS-Tn) and NT-proBNP—were measured using electrochemiluminescence immunoassays (ECLIA) on the Cobas e601 platform (Roche Diagnostics GmbH, Mannheim, Germany).

All post-PCI blood samples were collected in fasting state between 24 and 48 h after intervention.

Echocardiographic and laboratory data were transferred and cross-checked using Microsoft Excel (Microsoft Corporation, Redmond, WA, USA; version 2308) for consistency and missing values prior to analysis.

Renal function, assessed by estimated glomerular filtration rate (eGFR), and left ventricular ejection fraction (LVEF) were included as covariates in sensitivity analyses to account for potential confounding by kidney function and systolic performance. Medication data (e.g., ACE inhibitors, ARBs, β-blockers, statins, and diuretics) were not consistently recorded across the cohort and were therefore excluded from the multivariable models. This limitation was considered when interpreting associations between inflammatory markers and diastolic parameters.

### 2.4. Assessment of Diastolic Dysfunction

Diastolic dysfunction was graded according to the 2016 ESC/EACVI algorithm integrating four standard parameters: average E/e′ > 14, septal e′ < 7 cm/s or lateral e′ < 10 cm/s, left atrial volume index (LAVI) > 34 mL/m^2^, and tricuspid regurgitation (TR) velocity > 2.8 m/s. When parameters were discordant, classification followed the guideline decision rules and was adjudicated by two certified echocardiographers blinded to biomarker data. E/e′ values were averaged from septal and lateral measurements. LAVI was obtained by the biplane Simpson method and indexed to body surface area. TR velocity was measurable in 91% of patients; missing values did not influence grade allocation. To assess reproducibility, 20 random studies were re-evaluated, yielding κ = 0.86 for inter-observer agreement.

Transthoracic echocardiography was performed using a Vivid E9 ultrasound system (GE Healthcare, Chicago, IL, USA) equipped with an M5Sc-D transducer (1.5–4.6 MHz).

Image acquisition and quantification were performed according to the 2016 ESC/EACVI recommendations using EchoPAC software, version 203 (GE Healthcare, Chicago, IL, USA).

E/e′ values were averaged from septal and lateral measurements. LAVI was obtained by the biplane Simpson method and indexed to body surface area.

The degree of diastolic dysfunction was determined by transthoracic echocardiography performed by certified cardiologists, in accordance with the 2016 European Society of Cardiology (ESC) and European Association of Cardiovascular Imaging (EACVI) guidelines. Diagnostic classification was based on the integration of the following standard parameters:

- Average E/e′ ratio > 14;
- Septal e′ < 7 cm/s or lateral e′ < 10 cm/s;
- Left atrial volume index (LAVI) > 34 mL/m^2^;
- Tricuspid regurgitation (TR) velocity > 2.8 m/s.

Patients were classified as having normal diastolic function (grade 0) if ≤1 abnormal parameter was present. Grades 1 to 3 reflected increasing severity of dysfunction based on the number and combination of abnormal findings, following ESC criteria. This approach ensured consistent grading across the cohort and allowed reproducibility and comparability with other studies.

TR velocity was measurable in 91% of patients; missing values did not influence grade allocation.

To assess reproducibility, 20 random studies were re-evaluated, yielding κ = 0.86 for inter-observer agreement.

### 2.5. Statistical Analysis

All data were analyzed using SPSS software (Version 30, Armonk, NY, USA). Continuous variables were assessed for normality and presented as mean ± standard deviation (SD) or median and interquartile range (IQR), depending on distribution. Categorical variables were expressed as frequencies and percentages.

To assess the primary objective, ordinal logistic regression (proportional odds model) was employed, with the grade of diastolic dysfunction as the dependent variable. Predictors included hypertension, type 2 diabetes, dyslipidemia, obesity, and post-interventional inflammatory markers (leukocytes, neutrophils, and CRP). A *p*-value < 0.05 was considered statistically significant. Multicollinearity was tested (VIF < 2); proportional odds assumption was verified by Brant test (*p* = 0.43). Missing data < 5% were handled by complete-case analysis. A post hoc power analysis indicated 80% power to detect medium effect sizes (f^2^ = 0.06, α = 0.05).

The primary analysis used ordinal logistic regression (proportional odds model) with diastolic dysfunction grade (0–3) as the dependent variable and post-procedural inflammatory markers (leukocytes, neutrophils, and CRP) together with metabolic comorbidities (hypertension, type 2 diabetes, dyslipidemia, and obesity) as predictors. The proportional odds assumption was verified with the Brant test (*p* = 0.43). Model calibration was acceptable (Hosmer–Lemeshow χ^2^ = 6.21, *p* = 0.40); pseudo-R^2^ = 0.12. Cut-points corresponded to transitions between grades 1, 2, and 3. Adjusted odds ratios (aORs) and 95% CIs were reported for all predictors.

A secondary exploratory linear regression treated diastolic grade as a continuous outcome to assess the directionality of biomarker effects. Given the exploratory design, no multiplicity correction was applied; *p*-values were interpreted descriptively. Sensitivity analyses were performed (i) after collapsing grades 0–1 vs. 2–3 and (ii) restricted to patients with preserved EF (≥50%). All analyses were conducted in SPSS v30 (IBM, Armonk, NY, USA), with statistical significance set at two-tailed *p* < 0.05.

Sensitivity analyses adjusted for eGFR and LVEF were performed to account for potential confounding by renal function and systolic performance.

## 3. Results

### 3.1. Patient Demographics and Lifestyle Characteristics

The study cohort included a total of 181 patients diagnosed with acute coronary syndrome and treated interventionally. Of these, 65.7% were male (*n* = 119) and 34.3% were female (*n* = 62), indicating a male predominance consistent with the known epidemiology of coronary disease. The mean age of the patients was 62.8 ± 11.3 years, with an age range between 27 and 89 years, reflecting a typical distribution of middle-aged to elderly adults. The age distribution was symmetric (skewness = −0.020), without notable outliers or extreme values.

As shown in Table 1, the population was evenly distributed by residence (52.5% urban, 47.5% rural). Obesity (48.1%) and smoking (48.6%) were prevalent, indicating a high burden of modifiable risk factors. Alcohol consumption was uncommon (8.3%, *n* = 15), exhibiting a right-skewed distribution (skewness = 3.05; kurtosis = 7.39) consistent with predominantly abstinent behavior. A positive family history of cardiovascular disease was present in 13.8% (*n* = 25) of participants.

**Figure 1:**
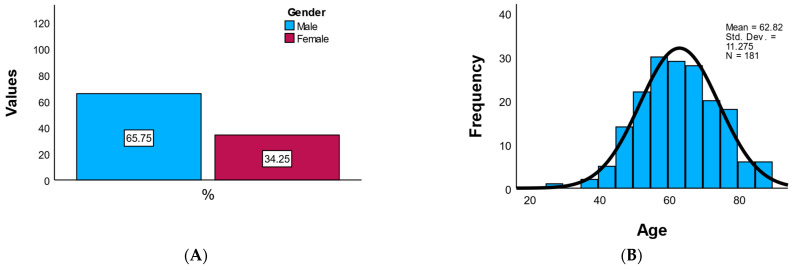
Distribution of Gender and Age in the Study Population. (**A**) Bar chart showing the proportion of male and female patients (*n* = 181), with counts and percentages displayed above each bar. The cohort demonstrates a male predominance (65.7%). (**B**) Histogram illustrating the age distribution of the study population, showing an approximately normal curve centered around 63 years.

Overall, the demographic profile reflects a high-risk cardiovascular population, predominantly male, with a high prevalence of obesity and smoking, and balanced geographic representation, presented in Figure 1. Figure 1A presents a bar chart of gender distribution, displaying the counts and percentages of male and female participants, while Figure 1B illustrates the age histogram.

### 3.2. Main Model Results

The model was globally significant, with a log-likelihood of −190.25 and an AIC of 400.5, indicating a good overall fit.

Among the predictors evaluated, C-reactive protein (CRP) approached statistical significance (*p* = 0.081) with a positive coefficient (*β* = 0.004), suggesting a potential weak association between systemic inflammation and a higher degree of diastolic dysfunction.

Hypertension also showed a positive coefficient (*β* = 0.44), although it did not reach statistical significance (*p* = 0.156). This may indicate a trend toward an association that warrants further validation in a larger sample.

**Figure 2:**
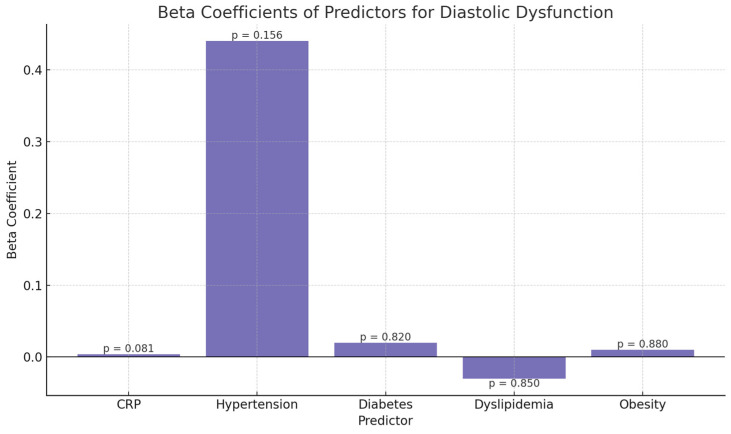
Beta Coefficients and Statistical Significance of Predictors for Diastolic Dysfunction. Bar plot illustrating the beta coefficients of clinical and inflammatory predictors in the ordinal logistic regression model assessing diastolic dysfunction severity. While none of the predictors reached statistical significance, C-reactive protein (CRP) and hypertension showed positive coefficients, suggesting potential trends. *p*-values are indicated above each bar.

Comorbidities such as type II diabetes mellitus, dyslipidemia, and obesity (Figure 2) were not significantly associated with the degree of diastolic dysfunction (all *p* > 0.8), suggesting a weaker or possibly indirect impact in this context.

The proportional odds model was significant overall (log-likelihood = −190.25, AIC = 400.5). CRP showed a weak, non-significant association with higher diastolic dysfunction grade (*β* = 0.004; aOR 1.004, 95% CI 0.999–1.010; *p* = 0.081). Hypertension demonstrated a positive but non-significant coefficient (*β* = 0.44; aOR 1.55, 95% CI 0.85–2.86; *p* = 0.16). Other metabolic comorbidities (type 2 diabetes, dyslipidemia, and obesity) were not independently associated (all *p* > 0.8).

In the sensitivity analysis collapsing grades 0–1 vs. 2–3, the direction and magnitude of coefficients were unchanged (CRP *p* = 0.09). Restricting the cohort to preserved EF yielded similar trends.

### 3.3. Leukocyte Markers

Post-interventional leukocyte and neutrophil values had positive but statistically insignificant coefficients (*p* = 0.466 and *p* = 0.762), suggesting that their variations are not directly predictive of the severity of diastolic dysfunction in the first 48 h.

**Figure 3:**
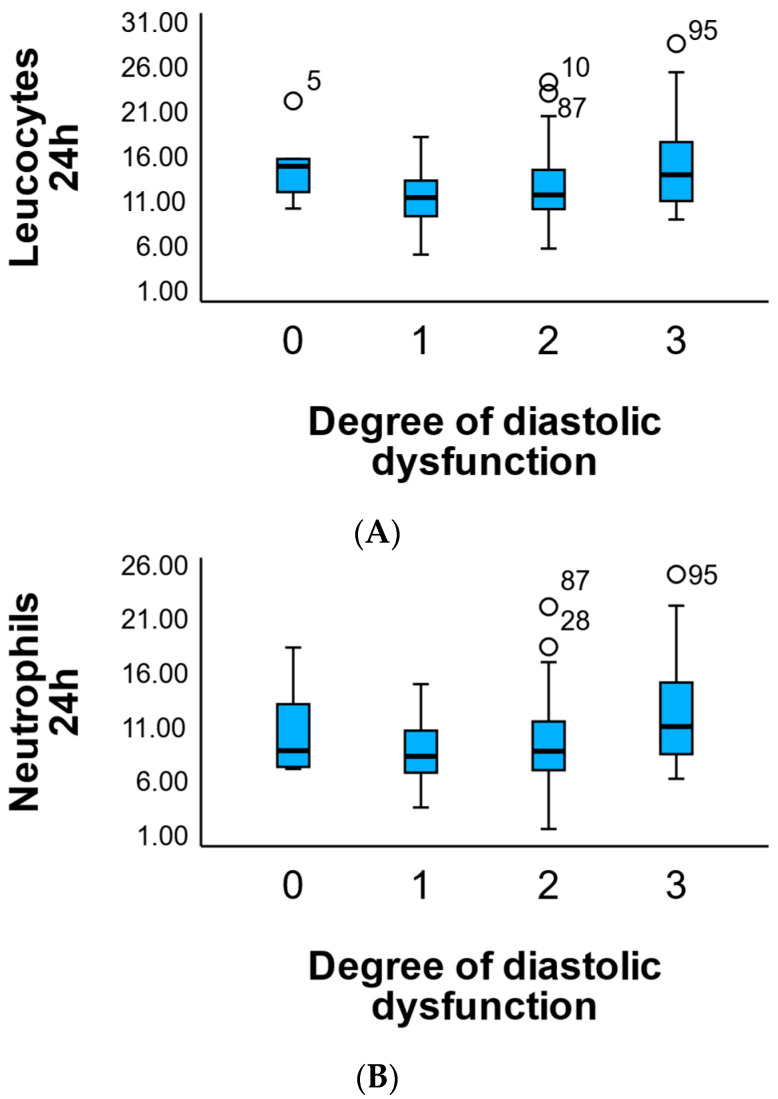
Distribution of Leukocyte and Neutrophil Counts by Degree of Diastolic Dysfunction. (**A**) Boxplot illustrating leukocyte levels (×10^3^/μL) at 24 h post-intervention, stratified by the degree of diastolic dysfunction (0–3). (**B**) Boxplot illustrating neutrophil levels (×10^3^/μL) at 24 h post-intervention, stratified by the degree of diastolic dysfunction (0–3). Outliers are marked above the upper whiskers.

As shown in Figure 3A, leukocyte counts at 24 h post-intervention tend to increase progressively with the severity of diastolic dysfunction. Although median leukocyte levels were relatively similar across grades 0 to 2, patients with grade 3 dysfunction exhibited visibly higher interquartile ranges and a greater number of outliers, suggesting elevated systemic inflammation in those with more advanced diastolic impairment.

Similarly, Figure 3B reveals a comparable pattern for neutrophil counts, with patients in grade 3 dysfunction showing the highest median and dispersion. Notably, while the lower degrees of dysfunction (0–2) maintained relatively stable neutrophil levels, grade 3 demonstrated a marked rise in inflammatory cell activity, as evidenced by the increased upper whiskers and extreme values.

These visualizations support the hypothesis that higher degrees of diastolic dysfunction are associated with amplified post-interventional inflammatory responses, particularly regarding innate immune activation as reflected by leukocyte and neutrophil counts.

### 3.4. Regression Analysis

The results of the regression analyses are presented in Table 2 and Table 3.

In the primary ordinal logistic regression model, C-reactive protein (CRP) showed only a weak, non-significant trend toward association with higher diastolic dysfunction grade (*β* = 0.004; adjusted OR 1.004, 95% CI 0.999–1.010; *p* = 0.081). Other predictors, including hypertension, diabetes, dyslipidemia, obesity, and sex, were not significantly associated with diastolic dysfunction severity. The proportional odds assumption was verified (Brant test *p* = 0.43), and overall model fit was modest (Pseudo-R^2^ = 0.12).

In the exploratory linear regression model, CRP remained positively correlated with diastolic dysfunction severity (*β* = 0.002; 95% CI 0.0005–0.004; *p* = 0.010), although the explained variance was low (R^2^ = 0.036). These findings suggest that elevated CRP levels may reflect a mild inflammatory response related to impaired relaxation rather than a strong independent determinant of diastolic dysfunction.

The results collectively indicate that while inflammatory markers show directional trends consistent with subclinical myocardial stress, their effect sizes are small and of limited predictive value in this post-PCI cohort. CRP remained borderline non-significant after adjustment (*β* = 0.004; 95% CI 0.999–1.010; *p* = 0.081).

### 3.5. Distribution of Diastolic Dysfunction

**Figure 4:**
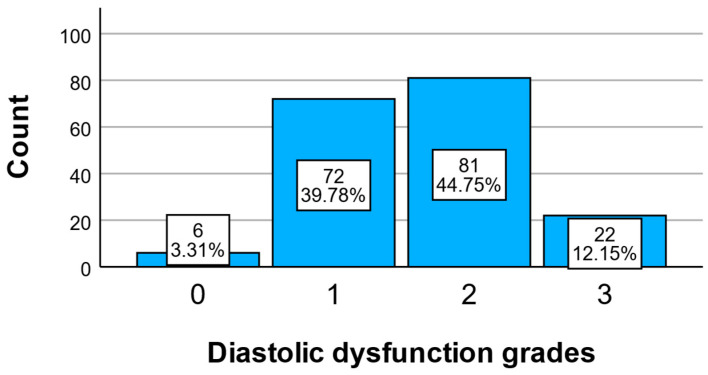
Distribution of diastolic dysfunction grades (0–3) in the study population.

To characterize the severity of diastolic function impairment in the study population, the distribution of diastolic dysfunction grades (grade 0–3) was analyzed, as illustrated in Figure 4. This shows that the majority of patients included in the study had mild to moderate forms of diastolic dysfunction:

- 39.78% were diagnosed with grade 1 dysfunction;
- 44.75% with grade 2.

Severe dysfunction (grade 3) was present in 12.15% of patients, while only 3.31% had normal diastolic function (grade 0). This distribution suggests an increased prevalence of diastolic impairment even in the early stages of acute coronary syndrome, reflecting a possible impact of inflammation and metabolic comorbidities on ventricular relaxation.

This classification serves as the basis for further analysis of associations between inflammatory markers and the severity of diastolic dysfunction. Of the total study population, the majority had mild (grade 1, 39.78%) and moderate (grade 2, 44.75%) dysfunction, together representing over 84% of cases. Only a small proportion of patients had normal diastolic function (grade 0, 3.31%) or severe dysfunction (grade 3, 12.15%), suggesting that this cohort is predominantly characterized by early to intermediate stages of diastolic impairment.

**Figure 5:**
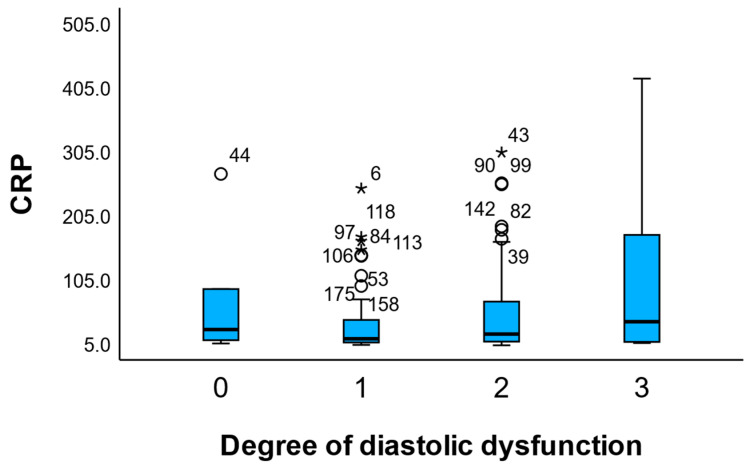
C-Reactive Protein (CRP) Levels According to the Degree of Diastolic Dysfunction. Boxplot showing the distribution of C-reactive protein (CRP) levels (mg/dL) across categories of diastolic dysfunction (grades 0 to 3). An upward trend in CRP concentrations is observed with increasing severity of diastolic impairment, indicating a possible association between systemic inflammation and advanced diastolic dysfunction. Outliers are marked individually above each box. NTrend shown for visualization purposes; association did not reach statistical significance (*p* = 0.081). Circles (○) indicate mild outliers (values between 1.5 and 3 times the interquartile range [IQR] from the nearest quartile), while asterisks (*) denote extreme outliers (values exceeding 3 × IQR).

In Figure 5, C-reactive protein (CRP) levels are displayed across the four grades of diastolic dysfunction. A progressive increase in median CRP values can be observed with worsening diastolic function, with the highest concentrations recorded in patients with grade 3 dysfunction. Although the variability also increased in this group, the overall upward trend suggests a possible link between systemic inflammation and more advanced diastolic impairment; however, the association did not reach statistical significance (*p* = 0.081).

### 3.6. Correlation Analysis Between Inflammatory Markers

Spearman correlation coefficients were calculated to assess the relationships between leukocytes, neutrophils, and C-reactive protein (CRP) measured 24 h post-intervention. The results are summarized in Table 4.

A strong positive correlation was observed between post-interventional leukocyte count and neutrophil count (*r* = 0.912, *p* < 0.001), indicating that neutrophils represent a major component of the overall leukocyte response following the procedure.

In contrast, CRP levels were moderately correlated with both leukocyte count (*r* = 0.257, *p* < 0.001) and neutrophil count (*r* = 0.267, *p* < 0.001), suggesting that while CRP elevation parallels cellular immune activation, it may also reflect additional systemic inflammatory pathways.

In addition, NT-proBNP correlated moderately with leukocyte count (ρ = 0.245), neutrophil count (ρ = 0.318), and CRP (ρ = 0.275), suggesting that inflammatory activation was associated with increased myocardial wall stress. A significant positive correlation was also observed between NT-proBNP and high-sensitivity troponin (TrHS 48 h; ρ = 0.232, *p* = 0.002), supporting a concurrent rise in biomarkers reflecting myocardial injury and hemodynamic load. CRP showed only a weak, non-significant relationship with troponin (ρ = 0.126, *p* = 0.091), consistent with its modest predictive role in the main analysis.

All correlations were statistically significant at the 0.01 level (two-tailed), confirming the internal consistency of inflammatory response markers in the acute post-interventional phase. The NT-proBNP–troponin correlation was descriptive and exploratory, not adjusted for confounders.

This table presents Spearman’s rho correlation coefficients between leukocyte count, neutrophil count, and C-reactive protein (CRP) measured at 24 h post-intervention. All correlations were statistically significant at the 0.01 level (2-tailed), indicating consistent inflammatory responses.

### 3.7. Exploratory Linear Regression

In the exploratory linear model, CRP remained positively associated with diastolic dysfunction (*β* = 0.002; 95% CI 0.0005–0.004; *p* = 0.010), although the model explained only 3.6% of variance (R^2^ = 0.036). This limited explanatory power indicates that, while CRP reflects inflammatory activity, it accounts for only a small portion of diastolic impairment severity. The results revealed the following:

A statistically significant positive coefficient for CRP (*β* = 0.002, *p* = 0.010) indicates that higher CRP levels are associated with more severe diastolic dysfunction.

However, the model explained only a small proportion of the variance in dysfunction (R^2^ = 0.036), suggesting that while CRP contributes significantly, its predictive value is limited.

**Figure 6:**
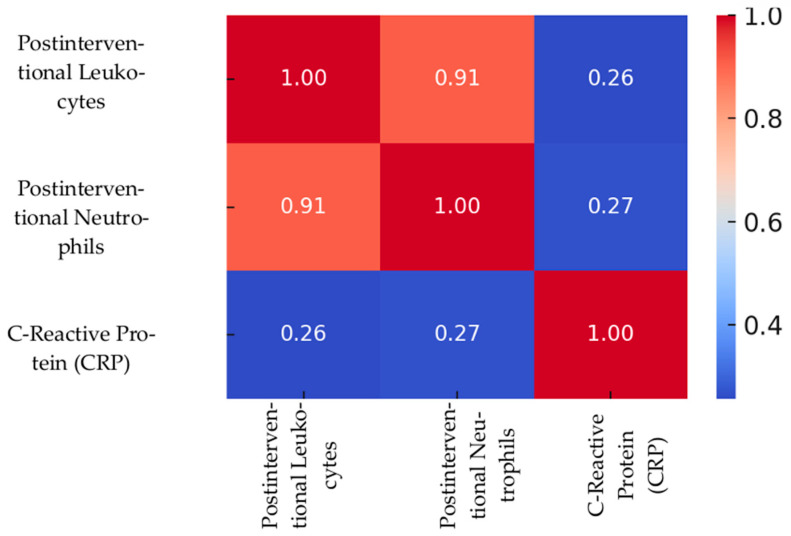
Spearman Correlation Heatmap Between Inflammatory Markers. Heatmap illustrating Spearman’s correlation coefficients between leukocyte count, neutrophil count, and C-reactive protein (CRP) measured 24 h post-intervention. A strong positive correlation was observed between leukocytes and neutrophils (ρ = 0.91), while both showed moderate correlations with CRP (ρ = 0.26–0.27), suggesting consistent activation of systemic inflammation pathways.

These findings support the notion that systemic inflammation—particularly elevated CRP—may serve as an early indicator of post-interventional diastolic dysfunction severity. Nonetheless, CRP alone is not sufficiently robust to function as a standalone clinical predictor. The heat map of Spearman correlation between inflammatory markers is shown in Figure 6.

## 4. Discussion

This study identified only a borderline association between post-procedural CRP and diastolic dysfunction severity after acute coronary syndromes. While CRP achieved significance in an exploratory linear model, the pre-specified ordinal model did not confirm a statistically robust effect. Therefore, CRP should be interpreted as an indicator of early post-PCI inflammation rather than a validated predictor of diastolic impairment [10,18,19,20]. The observed weak trend suggests that inflammation may accompany impaired relaxation without necessarily causing it. Inflammation may impair relaxation via oxidative stress, endothelial dysfunction, and microvascular remodeling.

Systemic inflammation appears to contribute to early impairment of diastolic function, possibly via oxidative stress, endothelial injury, and inflammatory cell infiltration. Previous studies have reported that elevated CRP levels are linked to myocardial stiffening and impaired ventricular relaxation—two key processes in the pathogenesis of diastolic dysfunction [21,22,23]. It should also be noted that only a small proportion of patients in our cohort presented with severe diastolic dysfunction (approximately 12%). Only a small proportion of patients in our cohort presented with severe diastolic dysfunction (about 12%), which likely reduced statistical power to detect significant effects of comorbidities such as diabetes, obesity, or dyslipidemia. These conditions are known to promote cardiac remodeling in larger, more heterogeneous populations.

An important consideration is the potential confounding role of systolic function. Although diastolic dysfunction is most often associated with preserved ejection fraction (EF), it can also occur in patients with reduced EF, particularly in the setting of myocardial injury and remodeling. Our cohort included individuals across the EF spectrum, which may have introduced heterogeneity in the observed associations. Sensitivity analyses restricted to preserved EF showed similar trends, suggesting that inflammation-related mechanisms operate irrespective of baseline systolic performance. Future studies limited to patients with preserved EF would help clarify whether these inflammatory associations are specific to the HFpEF phenotype or extend to those with systolic impairment.

The presence of metabolic comorbidities—hypertension, type 2 diabetes, dyslipidemia, and obesity—was not significantly associated with diastolic dysfunction severity in our multivariable model [24]. Although these conditions are recognized as contributors to cardiac remodeling and diastolic impairment, their impact may be modulated by factors such as disease duration, therapeutic control, or chronic inflammatory markers not included in the present analysis [25,26]. Furthermore, the relatively balanced distribution of these comorbidities within the study population may have limited the detection of significant differences, particularly given the smaller number of patients with severe dysfunction [27,28,29,30,31,32].

In addition to inflammatory markers, our analysis identified a significant correlation between NT-proBNP and high-sensitivity troponin at 48 h. This finding reflects the interplay between myocardial injury and hemodynamic wall stress, two mechanisms that often coexist in acute coronary syndrome. The release of troponin indicates myocyte necrosis, while NT-proBNP elevation reflects increased ventricular filling pressures and wall strain. Their correlation suggests that acute ischemic injury may amplify hemodynamic stress, which could partly explain the high prevalence of diastolic dysfunction observed in our cohort. Previous studies have shown that combined elevation of troponin and BNP identifies patients at greater risk of adverse remodeling and progression toward HFpEF, supporting the clinical relevance of this association.

The modest R^2^ (≈ 3.6%) underscores the limited explanatory capacity of inflammation alone. No meaningful interaction was observed between CRP and hypertension (*p* = 0.23), and stratification by ACS type (STEMI vs. NSTEMI) yielded consistent non-significant results. Collectively, these data highlight the multifactorial nature of early diastolic dysfunction, where inflammation likely acts in concert with ischemic injury and hemodynamic stress rather than as an independent determinant [33,34].

### 4.1. Study Limitations

The retrospective, single-center design may limit the generalizability of the findings.

The study lacked additional inflammatory markers (e.g., IL-6, TNF-α), which would have allowed a more nuanced characterization of systemic inflammation.

Diastolic dysfunction was graded using standard transthoracic echocardiography, without advanced imaging techniques (e.g., strain imaging and cardiac MRI).

Another limitation is the absence of systematic follow-up echocardiographic evaluations. Our analysis focused exclusively on the early post-interventional window (24–48 h), which provides insight into acute inflammatory and hemodynamic changes but does not capture the trajectory of diastolic function over time. Since diastolic dysfunction may improve, persist, or worsen in the weeks to months after PCI, depending on myocardial recovery and remodeling, longitudinal assessment is crucial. Future prospective studies with serial echocardiographic follow-up are needed to determine whether the observed associations with inflammation and biomarkers translate into sustained or progressive diastolic impairment.

Medication profiles (ACEi/ARB/ARNI, β-blockers, statins, and diuretics) were not consistently available and thus not included in multivariable models, which may have attenuated some associations.

The distribution of diastolic dysfunction severity was uneven, with relatively few patients in the severe dysfunction group. This imbalance may have limited the ability to detect significant associations with certain comorbidities.

Medication profiles (e.g., ACEi/ARB/ARNI, β-blockers, statins, and diuretics) and coronary lesion complexity were not systematically recorded and therefore could not be included in the multivariable model. Renal function was available only in a subset of patients. Baseline inflammatory marker values were incomplete, precluding analysis of Δ-changes. Echocardiography was consistently performed within 24–48 h post-PCI under stable volume status before diuretic titration. The analysis did not account for confounders such as renal function, anti-inflammatory medication use, or the duration of diabetes mellitus.

Additionally, several potentially relevant covariates were not systematically available across the dataset. Renal function (eGFR) and left-ventricular ejection fraction (LVEF) were incorporated in sensitivity analyses, yet other peri-procedural or treatment-related factors—such as Killip class, use of β-blockers, ACE inhibitors/ARBs/ARNIs, diuretics, and statins—could not be adjusted for because medication data were incomplete in the hospital records. The lack of serial biomarker measurements (baseline → 24–48 h) also prevented Δ-marker modeling, which may have captured dynamic inflammatory responses more accurately than single time-points. These omissions may have led to residual confounding and attenuation of true associations between inflammation and diastolic function. Future prospective studies should therefore include standardized collection of renal, hemodynamic, and pharmacologic variables, along with paired biomarker sampling, to refine risk adjustment and mechanistic interpretation.

### 4.2. Clinical Implications and Future Directions

These results underscore the importance of monitoring systemic inflammation during the early post-interventional period, especially in patients with metabolic risk factors. Future prospective studies with larger cohorts, comprehensive inflammatory profiling, and advanced imaging are warranted to validate CRP’s utility as a biomarker for early prediction of diastolic dysfunction following ACS.

The timing of assessment is crucial when interpreting the relationship between inflammation and diastolic dysfunction. Our study focused on the acute 24–48 h post-PCI window, a period during which systemic inflammation peaks and early complications are most likely to manifest. However, many patients experience subsequent remodeling, recovery, or late complications in the weeks to months after intervention. In this later phase, the role of inflammation may shift from acute injury signaling to chronic low-grade activation, which can drive progressive ventricular stiffening and adverse outcomes. Therefore, the clinical significance of inflammation needs to be evaluated across both acute and chronic settings, ideally through longitudinal follow-up with serial biomarker and echocardiographic assessments.

An unresolved question is whether the early association between systemic inflammation and diastolic dysfunction observed in our study translates into persistent or progressive impairment. If this relationship is confirmed in longitudinal studies, it could strengthen the rationale for considering targeted anti-inflammatory therapies as part of post-PCI management, particularly in metabolically vulnerable patients. Current evidence from other cardiovascular settings suggests that controlling systemic inflammation may improve ventricular compliance and reduce remodeling, but this remains to be validated in ACS patients post-PCI. Importantly, in our cohort, patients with active systemic infections were excluded, reducing the likelihood that infection-driven inflammation confounded our findings. Future longitudinal studies using advanced imaging and multi-marker panels are needed to validate these preliminary findings.

## 5. Conclusions

In this retrospective study, elevated C-reactive protein (CRP) levels measured 24–48 h post-intervention showed a non-significant trend toward association with a higher degree of diastolic dysfunction in patients with acute coronary syndrome (ACS). While leukocyte-based markers, such as total leukocyte and neutrophil counts, showed only weak or non-significant correlations, CRP consistently demonstrated an upward trend with increasing diastolic impairment, as confirmed by both ordinal and linear regression analyses.

Moreover, the presence of major metabolic comorbidities—such as hypertension, type 2 diabetes mellitus, dyslipidemia, and obesity—was not significantly associated with diastolic dysfunction severity in multivariable models. This suggests that acute systemic inflammation may play a more prominent role in early post-interventional cardiac impairment than chronic metabolic burden alone.

Elevated CRP measured 24–48 h after PCI showed only a weak, non-significant association with diastolic dysfunction in the primary model. While exploratory analysis suggested a possible trend, the overall explanatory power was low. Therefore, CRP may serve as a complementary—but not stand-alone—marker of early post-interventional cardiac stress. Future multicenter prospective studies integrating inflammatory, hemodynamic, and imaging parameters are warranted to validate these preliminary findings.

## Figures and Tables

**Table 1:** Demographic, Lifestyle, and Clinical Risk Characteristics of the Study Population.

Statistics		N	Mean	Std. Deviation	Skewness	Kurtosis	Minimum	Maximum
Gender	Male	181	119	65.7%	0.669	−1.570	1.00	2.00
	Female		62	34.3%				
Age (mean ± SD)			62.82	11.275	−0.020	−0.192	27	89
Environment	Urban		95	52.5%	0.100	−2.012	1	2
	Rural		86	47.5%				
Obesity	No		94	51.9%	0.078	−2.016	0	1
	Yes		87	48.1%				
Smoking	No		93	51.4%	0.056	−2.019	0	1
	Yes		88	48.6%				
Alcohol consumption	No		166	91.7%	3.051	7.393	0	1
	Yes		15	8.3%				
Family history	No		156	86.2%	2.115	2.502	0	1
	Yes		25	13.8%				

**Table 2:** Ordinal Logistic Regression Model for Predictors of Diastolic Dysfunction Grade.

Predictor	*β*	Adjusted OR (95% CI)	*p*-Value
CRP (mg/dL)	0.004	1.004 (0.999–1.010)	0.081
Hypertension	0.44	1.55 (0.85–2.86)	0.16
Type 2 Diabetes	0.21	1.23 (0.64–2.38)	0.53
Dyslipidemia	−0.07	0.93 (0.49–1.76)	0.84
Obesity	0.11	1.12 (0.58–2.15)	0.74
Age (per 10 years)	0.08	1.08 (0.96–1.22)	0.21
Sex (male)	−0.15	0.86 (0.45–1.63)	0.64

Pseudo-R^2^ = 0.12; Brant test *p* = 0.43.

**Table 3:** Exploratory Linear Regression Model for Diastolic Dysfunction Severity.

Predictor	*β* (95% CI)	*p*-Value
CRP (mg/dL)	0.002 (0.0005–0.004)	0.010
Hypertension	0.09 (−0.04–0.23)	0.18
Type 2 Diabetes	0.03 (−0.11–0.17)	0.64
Dyslipidemia	−0.02 (−0.16–0.12)	0.76
Obesity	0.04 (−0.10–0.18)	0.57
Age (per 10 years)	0.01 (−0.02–0.04)	0.29
Sex (male)	−0.06 (−0.19–0.08)	0.41

R^2^ = 0.036.

**Table 4:** Spearman Correlation Matrix Between Post-Interventional Inflammatory Markers.

Correlations			Leucocytes 24 h (×10^3^/µL)	Neutrophils 24 h (×10^3^/µL)	CRP (mg/dL)	NT-proBNP (pg/mL)	TrHS 48 h (ng/L)
Spearman’s rho	Leucocytes 24 h (×10^3^/µL)	rho	1.000	0.912 **	0.257 **	0.245 **	0.248 **
		*p*	.	<0.001	<0.001	<0.001	<0.001
	Neutrophils 24 h (×10^3^/µL)	rho	0.912 **	1.000	0.267 **	0.318 **	0.305 **
		*p*	<0.001	.	<0.001	<0.001	<0.001
	CRP (mg/dL)	rho	0.257 **	0.267 **	1.000	0.275 **	0.126
		*p*	<0.001	<0.001	.	<0.001	0.091
	NT-proBNP (pg/mL)	rho	0.245 **	0.318 **	0.275 **	1.000	0.232 **
		*p*	<0.001	<0.001	<0.001	.	0.002
	TrHS 48 h (ng/L)	rho	0.248 **	0.305 **	0.126	0.232 **	1.000
		*p*	<0.001	<0.001	0.091	0.002	.
		N	181				

CRP = C-reactive protein (mg/dL) measured post-intervention; N = number of patients; *p* = statistical significance; rho = Correlation Coefficient. ** Correlation is significant at the 0.01 level (2-tailed). The dot (.) denotes cases where the variable was correlated with itself (rho = 1.000) or where a *p*-value is not applicable.

## Data Availability

All the data processed in this article are part of the research for a doctoral thesis, being archived in the database of the corresponding author, where the interventions were performed.
